# Supplementary material for: Non-invasive eye tracking and retinal view reconstruction in free swimming schooling fish
Source: Commun Biol. 2024 Dec 12;7:1636. doi: 10.1038/s42003-024-07322-y (PMC11638265; doi:10.1038/s42003-024-07322-y)
Supplement: Supplementary file 2 — Supplemental material [file 42003_2024_7322_MOESM2_ESM.pdf]

Supplementary Information for Non-invasive eye tracking and retina view reconstruction in free swimming schooling fish

Ruiheng Wu[1,2], Oliver Deussen[1,2], Iain D. Couzin[2,3,4*], and Liang Li[2,3,4*]

[1] *Department of Computer and Information Science, University of Konstanz, 78464, Konstanz, Germany*

[2] *Centre for the Advanced Study of Collective Behaviour, University of Konstanz, 78464 Konstanz, Germany*

[3] *Department of Collective Behaviour, Max Planck Institute of Animal Behavior, 78464 Konstanz, Germany*

[4] *Department of Biology, University of Konstanz, 78464 Konstanz, Germany*

1 Dataset

The current datasets of fish body mesh or pose, such as Fish-100, AdamPose [1], and FishNet [2], are not sufficient for our eye-tracking purposes. This is because they are limited to single-fish models and lack labeling for eye movement. To achieve our goal of reconstructing and analyzing a fish's visual attention in a multi-fish scenario, we created our own dataset for all training and evaluation processes.

Published data and code The public dataset includes the coordinates of fish and their eye movements as a CSV file, which we used for statistical analysis, along with a pair of videos as an example of input data. The public code repository contains the fish 4D reconstruction module (DeepShapeKit) and the fish eye tracking modules, enabling users to obtain data on fish swimming poses and eye movements.

In addition to the reconstruction and tracking code, the trained weights for the neural networks involved (Mask R-CNN and DeepLabCut) are included, allowing users to process our shared videos directly. However, users intending to apply our code to other fish recordings may need to retrain the networks with data tailored to their specific input scene, fish species, and view angle, as well as calculate the projection matrix for the recording camera.

We also provide a demo script that processes the demo videos to extract eye movement data without requiring manual intervention.

2 Procedure of generating eye detection evaluation data

We provide here the details of generating eye detection evaluation data. The idea is drawing fish eye images with randomized pupil size and position, then synthesize a fish image with the generated fish eye by placing the eye image on the recorded fish body. Afterwards, we present the synthesized fish with generated fish eye to our eye tracking framework and compare the tracking result with the data we used for generating eye image. We prepare the data with the following steps as plotted in Supplementary Fig. 3:

1. Initialize a squared area with width w filled with grey color. This is similar to the fish skin color in our video recording.

2. Draw a light color filled circle at the center of the square with a radius of $\frac{w*2}{5}$ representing the fish eye area.

3. Draw a dark color filled circle with radius r_p at position (x_p, y_p). The radius r_p is sampled uniformly from $[\frac{w}{6} - \frac{w}{20}, \frac{w}{6} + \frac{w}{20}]$, and the coordinate x_p and y_p are both picked uniformly from $[\frac{w}{2} - \frac{w}{8}, \frac{w}{2} + \frac{w}{8}]$.

4. Blur the eye image with an average kernel of size $\frac{w}{6}$.

5. Place the blurred eye image on a fish image cropped from a video frame, and align the eye image to the fish body direction.

3 Evaluation results

In Supplementary Table 2, we present the detection error in the evaluation data. The generated eye images have a height and width of 160 pixels. Limited by the rasterized pixel image, the coordinates and radius of circles are all natural numbers. From the result, we can observe that the position detection has a relatively lower error, resulting in high precision in view angle. However, the detected radius of the pupil tends to be smaller than the ground truth value. This is due to the Gaussian blur effect, which reduces the circle area that can be identified as black. Since the crucial part for obtaining the fish's view angle is the position rather than the size, we will not attempt to reduce the pupil size detection error.

4 Eye Camera view direction based on detected eye movement

To let the eye camera simulate the fish's eye movement, the final step is adjusting its orientation frame by frame. The camera will start with the normal direction of the eye area on the 3D model, which is the view direction of no eye movement. We then map the detected 2D eye coordinate to the 3D view direction by setting an x and y direction relative to the fish model and rotate the virtual camera according to the 2D position as described in Supplementary Fig. 4.

$$C = N + x \cdot r_{goldfish} \cdot U_x + y \cdot r_{goldfish} \cdot U_y \tag{1}$$

Eq. 1 shows how we calculate the eye camera view direction C with the normal vector of the eye area N, the 2D eye coordinates x and y, and direction vectors on x and y axis. The direction vector represents the eye movement on x axis $r_{goldfish} \cdot U_x$ is calculated according to the goldfish

eye anatomy [3, 4]. We plot the eye structure of the goldfish in Supplementary Fig. 5. The view direction points from the retina center (green dot) to the pupil center (the intersection point of the view direction and cornea). The parameter $r_{goldfish}$ is the ratio between eye depth (blue line) and eye radius (half of the red line). On x axis, we add the normal vector N with the amount of movement on x axis times the the unit vector U_x. The same operation is performed on U_y, which gives us the eye movement on y axis. Adding the two vectors can show us the moving direction of the eye camera.

References

1. Wu, X., Huang, J., Wang, Y. & Wang, L. Pose estimation-based experimental system for analyzing fish swimming. *bioRxiv* (2023).
2. Sun, S., Pang, J., Shi, J., Yi, S. & Ouyang, W. Fishnet: A versatile backbone for image, region, and pixel level prediction. *Advances in neural information processing systems* **31** (2018).
3. Lee, S. & Stevens, C. F. General design principle for scalable neural circuits in a vertebrate retina. *Proceedings of the National Academy of Sciences* **104,** 12931–12935 (2007).
4. Claus, I. *et al.* Comprehensive optical design model of the goldfish eye and quantitative simulation of the consequences on the accommodation mechanism. *Vision Research* **154,** 115–121 (2019).

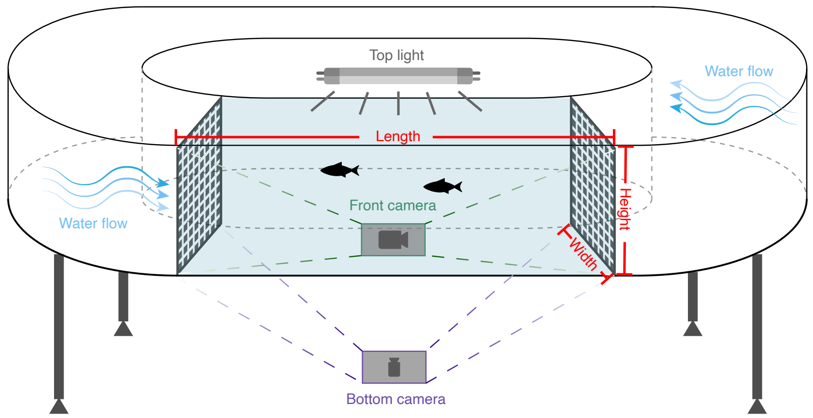

SUPPLEMENTARY FIGURE 1. Flow tank setup for recording fish swimming scenes. Water flow is generated by an engine located at the top right side of the circle. Two fish are confined within a swimming area measuring 88 cm in length, 25 cm in width, and 25 cm in depth using two grid plates that allow water to flow through. An area light is positioned above the flow tank to provide uniform illumination. Two cameras are placed at the front and bottom sides to capture the fish swimming scenes.

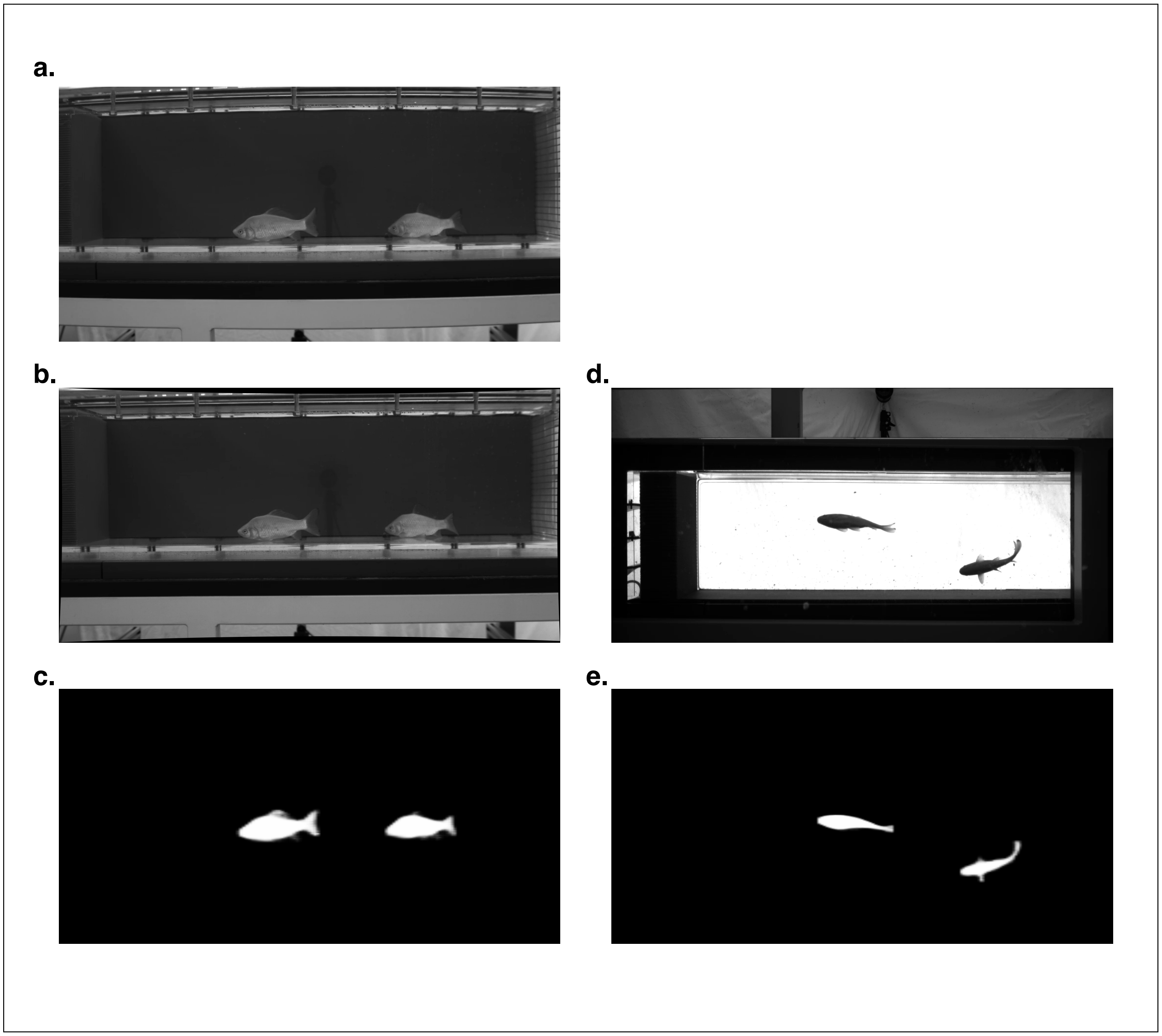

SUPPLEMENTARY FIGURE 2. Example of front and bottom image input. **a** Original front input with radial distortion. **b** Front input after correcting the distortion. **c** Front fish mask detected by Mask R-CNN. **d** Bottom input. **e** Bottom fish mask detected by Mask R-CNN.

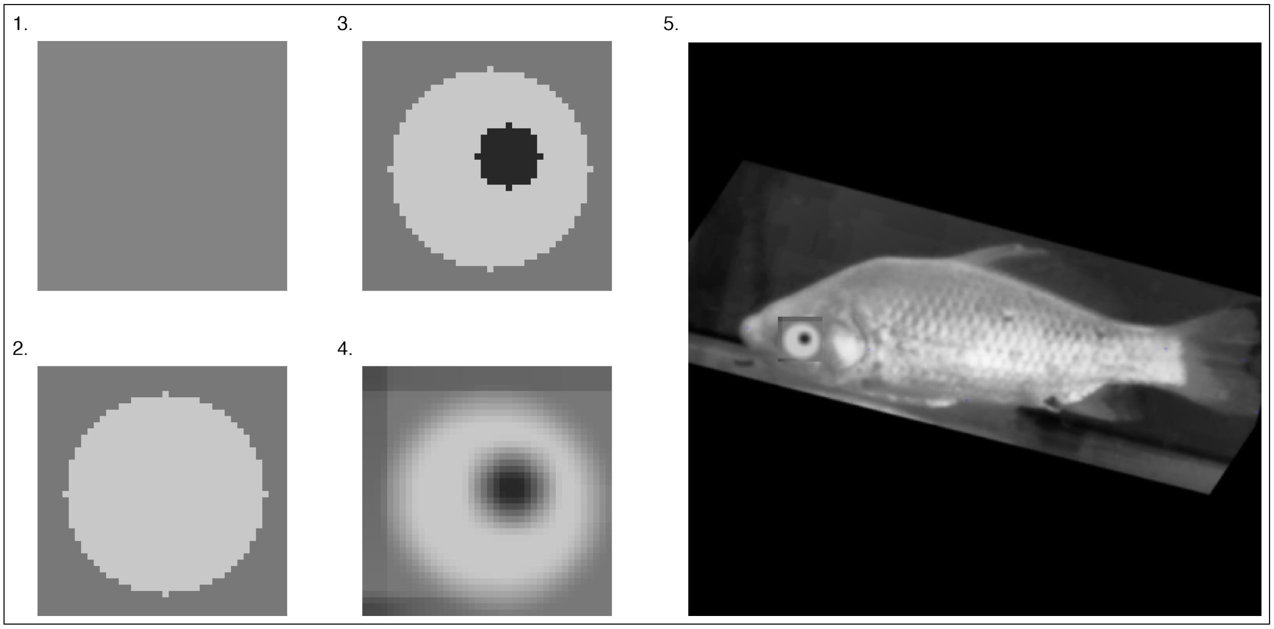

SUPPLEMENTARY FIGURE 3. Steps of generating evaluation data. 1. Grey background simulates fish's skin around eye. 2. The added light area represents the fish's eye sclera. 3. The added dark area represents the fish's pupil. 4. A Gaussian blur is applied to smooth the transition between areas, simulating the appearance of a real fish's eye.

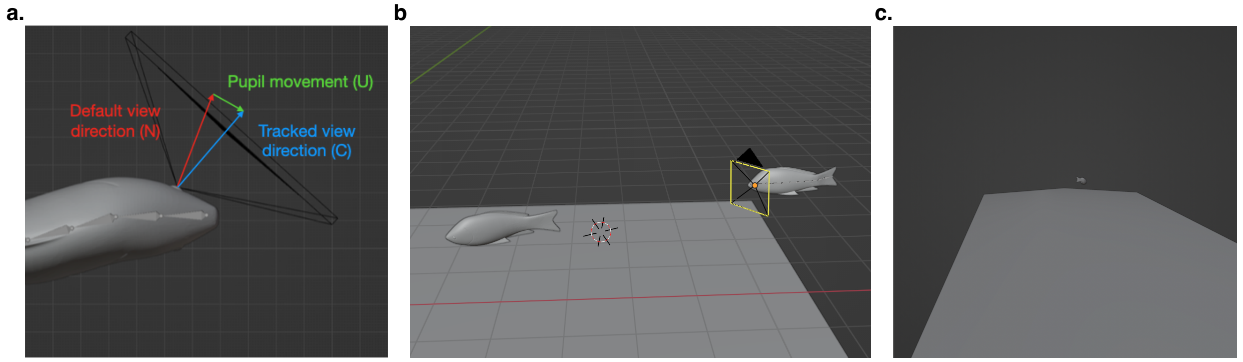

SUPPLEMENTARY FIGURE 4. Illustration of the retina view reconstuction. **a** Adjust camera view direction based on pupil movement. **b** Relative position of two fish in the example. **c** Example of captured image with fish eye camera.

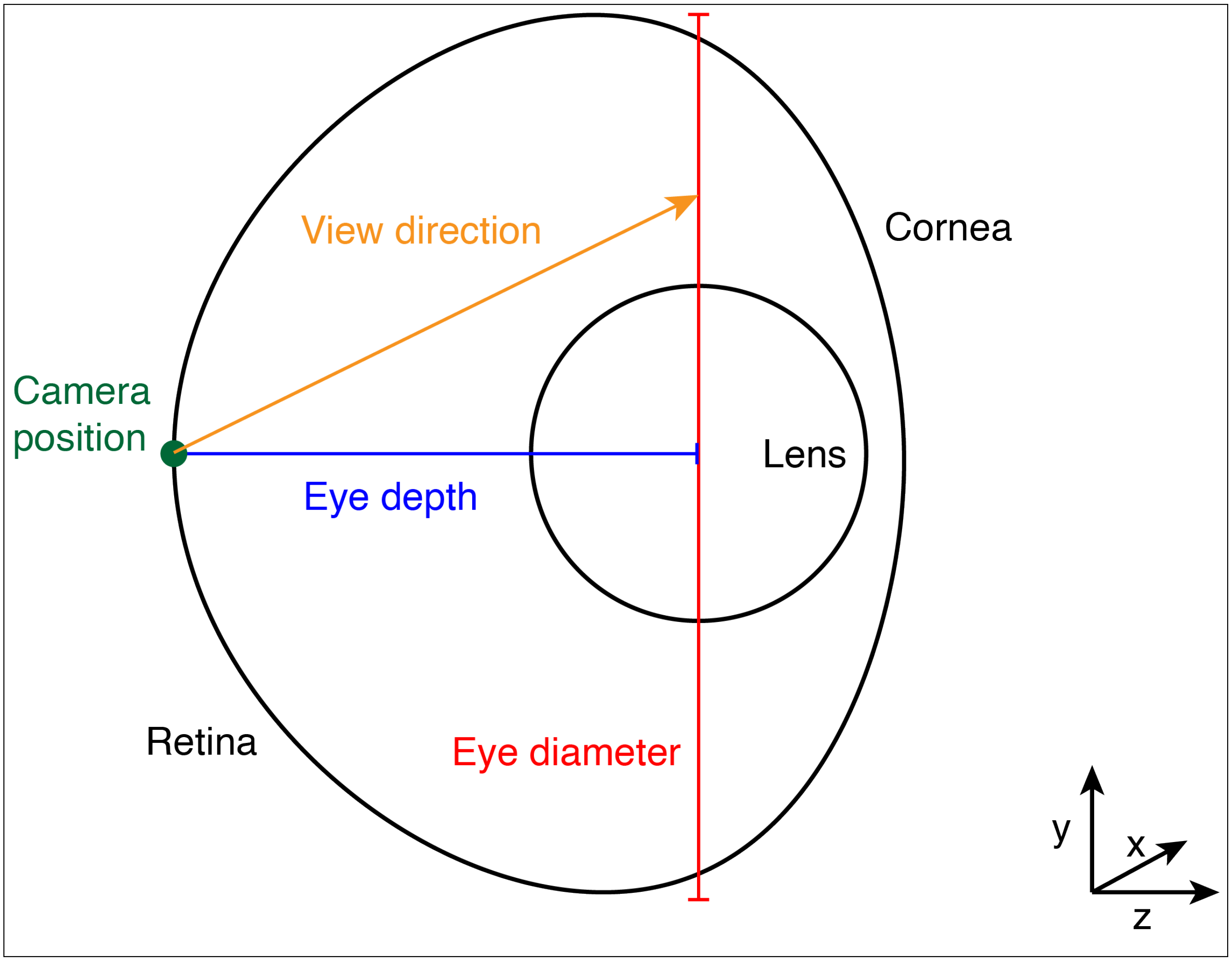

SUPPLEMENTARY FIGURE 5. The structure of the goldfish eye cut in half and the camera position on the model. The left side of the red line represents the retina, while the right side represents the cornea. The camera is placed at the center of the retina, indicated by the green dot. When the fish looks in another direction, its pupil moves along the red line. As the pupil moves, we adjust the camera to look in the direction from the green dot to the pupil.

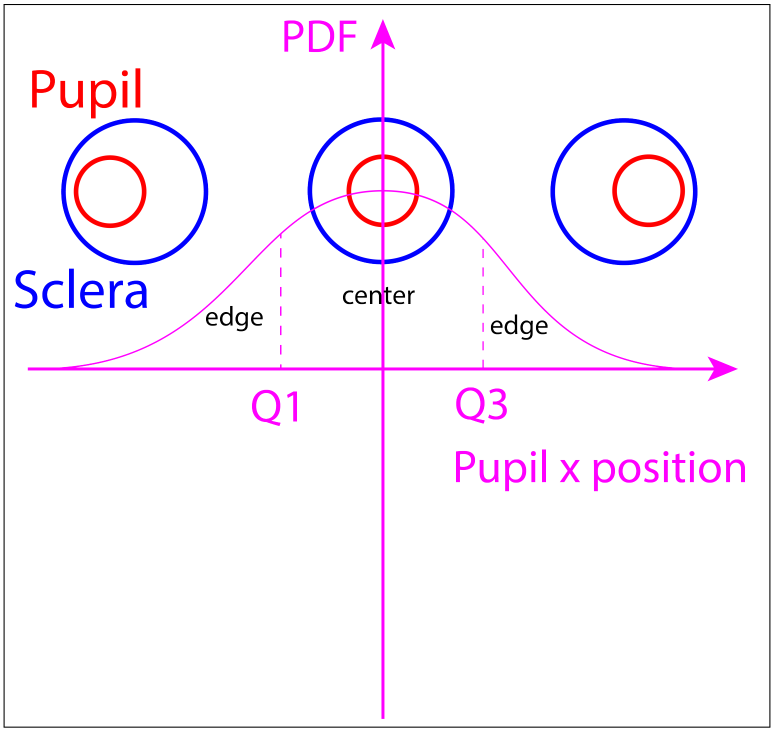

SUPPLEMENTARY FIGURE 6. Illustration of pupil position in edge cases and center cases. The horizontal position of the pupil is plotted along the x-axis, ranging from left to right, while the y-axis represents the probability density function (PDF) of the pupil's horizontal position. The split between edge and center cases is determined based on statistical analysis: pupil positions falling between the first and third quartiles are defined as center cases, while the remaining positions are classified as edge cases.

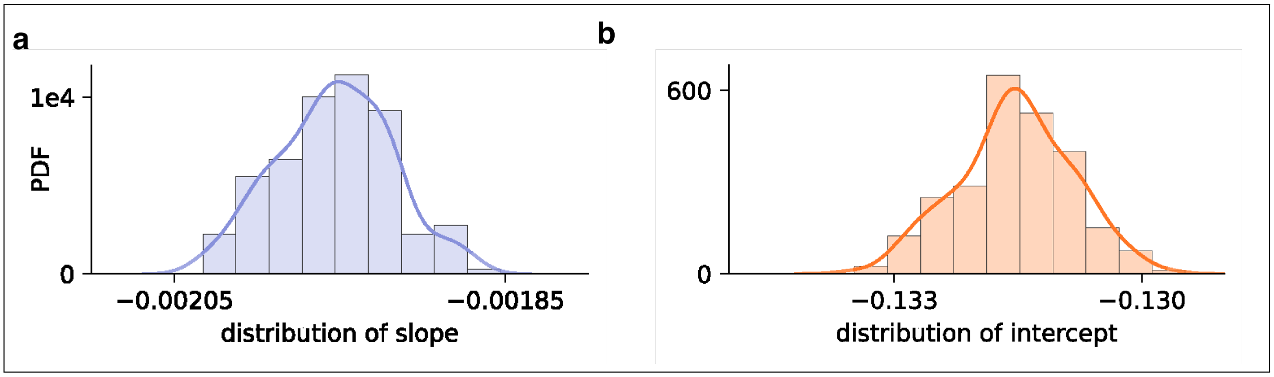

SUPPLEMENTARY FIGURE 7. Distribution of the slope and intercept when performing 200 iterations of sampling 6,000 points in the regression analysis. The sampled points contains 3,000 from the 37,639 cases where the leader was in the front-left position and 3,000 from the 7,860 cases where the leader was in the front-right position.

Table 1: Standard deviation of normalized front fish position in meters. The x-axis and y-axis are referring to Figure 5 in the main text.

	Dynamic	Static	Random
x-axis	0.0786 mm	0.0951 mm	8.638 mm
y-axis	0.0251 mm	0.0293 mm	0.862 mm

Table 2: Detection error on 110 manually labelled images and 500 synthesized eye images

	Manual Label			Synthesized		
	Angle	Pupil	Eye sclera	Angle	Pupil	Eye sclera
View angle mean error	$-2.45°$	-	-	$-0.10°$	-	-
View angle standard deviation	$2.65°$	-	-	$2.95°$	-	-
x axis mean error	-	-0.07 mm	0.04 mm	-	-0.09 mm	-0.02 mm
x axis standard deviation	-	0.12 mm	0.12 mm	-	0.33 mm	0.63 mm
y axis mean error	-	0.03 mm	-0.15 mm	-	-0.23 mm	-0.85 mm
y axis standard deviation	-	0.26 mm	0.57 mm	-	0.13 mm	1.19 mm

Table 3: Detection error comparison between edge cases (Q1 & Q3) and center cases (Q2) in manually labeled data

	View angle		Pupil position	
	Q1 & Q3	Q2	Q1 & Q3	Q2
mean error	-2.42°	-2.47°	-0.08 mm	-0.06 mm
standard deviation	3.02°	2.23°	0.11 mm	0.10 mm
minimum error	-8.95°	-8.54°	-0.32 mm	-0.31 mm
maximum error	4.62°	2.27°	0.12 mm	0.10 mm

Table 4: Detection error comparison between edge cases (Q1 & Q3) and center cases (Q2) in synthesized data

	View angle		Pupil position	
	Q1 & Q3	Q2	Q1 & Q3	Q2
mean error	-0.43°	0.31°	-0.17 mm	-0.01 mm
standard deviation	3.16°	2.70°	0.30 mm	0.34 mm
minimum error	-9.38°	-5.01°	-1.03 mm	-0.84 mm
maximum error	7.96°	7.06°	0.51 mm	0.90 mm

Table 5: A summery of shared data

Data	Description
Body posture reconstruction	code for reconstruction use DeepShapeKit
Eye tracking	code for extracting eye movement
Data analysis	code for statistical analysis
Sample videos	a pair of video clips for running the demo
Trained network	trained weights for the Mask-RCNN and DeepLabCut
