# Supplementary material for: Non-invasive eye tracking and retinal view reconstruction in free swimming schooling fish
Source: Commun Biol. 2024 Dec 12;7:1636. doi: 10.1038/s42003-024-07322-y (PMC11638265; doi:10.1038/s42003-024-07322-y)
Supplement: Supplementary file 3 — Description of Additional Supplementary Files [file 42003_2024_7322_MOESM3_ESM.pdf]

Description of Additional Supplementary Files

File name: Supplementary Video 1

Description: video summary of our pipeline
